# Co‐Existence Slows Diversification in Experimental Populations of *E. coli* and *P. fluorescens*

**DOI:** 10.1111/1462-2920.70061

**Published:** 2025-02-23

**Authors:** Gareth Howells, Aysha L. Sezmis, Christopher Blake, Michael J. McDonald

**Affiliations:** ^1^ School of Biological Sciences Monash University: Clayton Clayton Victoria Australia

**Keywords:** coexistence, experimental evolution, microbial ecology

## Abstract

Microbes grown in heterogeneous laboratory environments can rapidly diversify into multiple, coexisting variants. While the genetic and evolutionary mechanisms of laboratory adaptive radiations are well studied, how the presence of other species alters the outcomes of diversification is less well understood. To test the effect of co‐culture growth on the *Pseudomonas fluorescens* SBW25 adaptive radiation, *Escherichia coli* and *P. fluorescens* were cultured in monoculture and co‐culture for 8 weeks. In *P. fluorescens* monoculture, Wrinkly and Smooth Spreader types rapidly evolved and were maintained over 8 weeks, while *E. coli* monocultures evolved two colony types, a big and a small colony variant. In contrast, we found that in co‐culture, *E. coli* did not evolve small colony variants. Whole genome sequencing revealed the genetic basis of possible co‐culture specific adaptations in both *E. coli* and *P. fluorescens.* Altogether, our data support that the presence of multiple species changed the outcome of adaptive radiation.

## Graphical abstract

*Escherichia coli* and *Pseudomonas fluorescens* were cultured in monoculture and co‐culture for 8 weeks. Both species diversified in monoculture, while sequencing revealed mutations in *P. fluorescens* that were enriched in co‐culture with *E. coli* . A sequencing‐based invasion assay of 16S amplicons revealed that mutations in the PFLU_1661 gene confer the capacity to invade the *P. fluorescens* wild type.

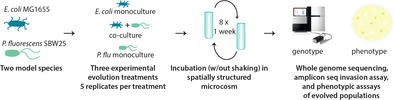

## 1 Introduction

The evolutionary diversification of a population into multiple, phenotypically distinct subpopulations that are each adapted to a new niche—adaptive radiation—is the fundamental process underlying the diversity of life (Schluter 2000). Adaptive radiations are driven by ecological opportunity, where genotypes that can access resources unavailable to other genotypes gain an evolutionary advantage. If a new niche specialist has a reduced capacity to utilise the resources of the progenitor population, the two types can coexist (Saxer et al. 2010). A well‐studied model of microbial adaptive radiation is the diversification of the bacterium *P. fluorescens* SBW25 into multiple distinct ecotypes, each adapted to a different niche in a spatially structured laboratory microcosm (Rainey and Travisano 1998; Kerr et al. 2002; Bantinaki et al. 2007; McDonald et al. 2009; Lind et al. 2015). The spatial structure is caused by the incubation of the microbial cultures without shaking. The lack of shaking allows cells producing a cellulose‐rich biofilm (Spiers et al. 2003) to attach to the glass walls of the test tube and co‐adhere into mats at the meniscus (Moshynets et al. 2022), and without mixing, an oxygen gradient builds in the tube (Kuśmierska and Spiers 2016). The two most readily identified ecotypes are the Smooth Spreader (SM), which has an agar plate colony morphology similar to the *P. fluorescens* SBW25 ancestor and inhabits the liquid column, and the Wrinkly Spreader (WS). The WS types (Ferguson et al. 2013; Lind et al. 2017) arise from mutational activation of diguanylate cyclases (DGCs) that cause overproduction of the second messenger c‐di‐GMP (McDonald et al. 2009; Goymer et al. 2006), overproduction of an acetylated cellulose polymer (Spiers et al. 2003, 2002) and ultimately formation of a self‐supporting microbial mat (Lind et al. 2019). The WS colonises the air–liquid interface and when grown on solid agar forms a distinctive wrinkled colony morphology. The WS and SM types evolve and are maintained by strong intraspecific competition for access to the resources in these niches (Rainey and Rainey 2003; Koza et al. 2017). In addition to the *P. fluorescens* SBW25 adaptive radiation, incorporating a spatial structure into laboratory evolution of several different species, including other *Pseudomonads* (Mukherjee et al. 2022), *Saccharomyces cerevisiae* (Frenkel et al. 2015) and *Burkholderia cenocepacia* (Traverse et al. 2013), has been shown to consistently promote the evolution of distinct, coexisting genotypes.

These studies provide important insights into evolutionary genetics and microbial adaptation to a new niche. However, most microbial species co‐exist with other species in complex communities, and a single species is unlikely to be able to colonise a new environment without competition from other species. Why might we expect the colonisation of a heterogeneous environment to be different with multiple competing species (Ghoul and Mitri 2016; Meroz et al. 2021)? One cause of evolutionary diversification in microbial populations is eco‐evolutionary feedback (Koeppel et al. 2013; Ponciano et al. 2009). In an environment with two species utilising resources, occupying space, as well as excreting and secreting molecules, these alterations to the environment can create new evolutionary pressures for both species. The fact that evolutionary change can happen on a similar timescale as ecological change has been one of the more surprising results of microbial evolution experiments. For instance, eco‐evolutionary feedback has been observed in experimental populations of *E. coli* that evolve cross‐feeding subpopulations that utilise acetate, a byproduct of rapid but inefficient glucose utilisation (Friesen et al. 2004). The evolution of WS, which evolves in a highly spatially structured environment, also depends on eco‐evolutionary feedback, since the founding *P. fluorescens* cells must consume oxygen, contributing to the oxygen gradient that provides the ecological opportunity for the WS (Koza et al. 2017).

Adaptive radiations depend upon the availability of an alternative niche—in the case of the WS, the air–liquid interface—and strong intraspecific competition for resources. The reliable evolution of WS types could partially be explained by the regularity of the abiotic environment, which provides the same strong diversifying selection. The presence of a second competing species could fundamentally change these conditions (Barraclough 2015). First, the second species may already occupy part of the alternative niche, reducing or altering the selective forces and thereby precluding (Calcagno et al. 2017) or altering evolutionary diversification (Jousset et al. 2016). Second, if the resource use of the second species overlaps with the first species, this may drive directional evolution of the two species to utilise different resources, thereby preventing the diversification of one or both of the species (Foster and Bell 2012; Osmond and de Mazancourt 2013).

Studies of co‐evolving laboratory populations of microbial species have shown that interspecies interactions can alter (Barber et al. 2021; Zhang et al. 2012; Gómez and Buckling 2013; Chu et al. 2021) and constrain evolutionary outcomes (Lawrence, Fiegna, et al. 2012), and that the presence of multiple species changes the outcomes of the *P. fluorescens* adaptive radiation (Chu et al. 2021). However, the impact of interspecies interaction on the genetic evolution of both *P. fluorescens* and the co‐evolving species remains an open question. To study the impact of co‐culture on the outcomes of *P. fluorescens* and *E. coli* adaptive radiations, we propagate populations of *P. fluorescens* and *E. coli* in monoculture and co‐culture and identify the phenotypic and genetic changes that evolve in each species.

## 2 Methods

### 2.1 Strains and Media

We used *E. coli* MG1655 K‐12 F–λ– ilvG– rfb‐50 rph‐1 and *P. fluorescens* SBW25. All strains were grown in King's B (KB) media (King et al. 1954), per litre; 20 g of proteose peptone No. 3, 10 mL of glycerol, 1.5 g of MgSO4 and 1.5 g of K2HPO4. The *E. coli* ancestor, small colony variant, and the *P. fluorescens* ancestor and WS variant are visually distinguishable on KB agar plates. After the 8‐week evolution experiment, new colony types were isolated from agar plates of each population, from each replicate, and stored at –80°C in glycerol stocks; these clones were used in subsequent experiments and DNA sequencing.

### 2.2 Evolution Experiment Protocol

Single colonies of ancestral *P. fluorescens* and *E. coli* were obtained by streaking out glycerol stocks onto agar plates. From these plates, a different single colony was used to found each replicate microcosm of *P. fluorescens* (replicates P1‐P5), *E. coli* (replicates E1‐E5) and *P. fluorescens* and *E. coli* co‐culture (replicates C1‐C5), with five replicates for each treatment. Microcosms were grown in 3 mL of liquid KB medium in 15 mL glass test tubes. We utilised 15 mL test tubes with a diameter of 13.8 mm, which are narrower than the 6 mL flat‐bottomed Bijou McCartney Bottles with a diameter of 22.5 mm used in the classic WS experiments (Rainey and Travisano 1998). Due to the narrower diameter of our test tubes, the ratio for the point of attachment (circumference) to total mat area is ~50% greater for our system, and the WS mats appear to be robust for the length of our experiment. After 7 days of incubation without disturbance in a 28°C incubator, tubes were vortexed to break down mats at the air–liquid interface. In a laminar flow and using filter tips, 3 μL of culture was transferred into 3 mL of fresh media (a 1000‐fold dilution). Every week, after vortexing, glycerol stocks were made of every culture and frozen at –80°C. This protocol was continued for 8 weeks (8 cycles). Fortnightly, populations were plated to check for contamination. Replicate population two from the *P. fluorescens* monoculture treatment was found to be contaminated during colony checks and so was not included in subsequent phenotypic and genetic analyses. We measured the absolute population sizes of *P. fluorescens* and *E. coli* in monoculture and co‐culture, finding that *P. fluorescens* population sizes were slightly lower in co‐culture at the conclusion of the evolution experiment ( *P. fluorescens* monoculture 1.05 ± 0.164 × 10^9^, coculture; 1.56 ± 0.635 × 10^8^ CFU/mL). The population size of *E. coli* is similar in monoculture and co‐culture (2.53 ± 0.516 × 10^9^, co‐culture 0.940 ± 0.441 × 10^9^CFU/mL). Every week, cultures were well mixed by vortexing and diluted by 10^3^‐fold. This method ensures an effective population size of 10^6^ for populations in monoculture and 10^5^ for populations in co‐culture. In these conditions, selection can discern beneficial mutations with a very small fitness effect in all treatments (~^1^/_N_) (Lachapelle and Colegrave 2015).

### 2.3 Colony Counts

Serial dilutions of each culture were prepared and 50 μL of the 10^−6^ dilution was plated onto KB agar. On agar, *E. coli* colonies were found to grow more rapidly than *P. fluorescens* and could overgrow *P. fluorescens* colonies if left in the incubator. To overcome this, emergent *E. coli* colonies were counted after 24 h in a 28°C incubator before plates were placed in a 6°C fridge; at this temperature, *E. coli* growth is arrested (Strocchi et al. 2006) and *P. fluorescens* can grow (Meng et al. 2017; Kahli et al. 2022). After 72 h, the proportions of *P. fluorescens* SM and WS were counted to provide information on community composition over the course of the experiment. *P. fluorescens* WS and SM colonies appeared green and were therefore readily distinguishable from *E. coli* colonies—which appeared beige and round—on agar plates.

### 2.4 Motility Assays

Swimming motility assays were performed in KB plates with 0.3% (w/v) agar. Plates were stabbed with 1 μL of OD_600_ 0.01 overnight culture. Diameter was measured after 24 h of growth at room temperature (22.5°C ± 2.5). Assaying at room temperature reduces evaporation and disturbance (Pentz and Lind 2021). Each clone (*n* = 5 large colony variant (LCV), small colony variant (SCV) *E. coli* , *n* = 4 *P. fluorescens* monoculture WS, SM, *n* = 5 WS, SM, *E. coli* from co‐culture) was measured in triplicate by inoculating from the same culture three times.

### 2.5 Biofilm Assays

Clones (*n* = 5 LCV, SCV *E. coli* , *n* = 4 *P. fluorescens* monoculture WS, SM, *n* = 5 WS, SM, *E. coli* from co‐culture) were grown for 7 days in 3 mL of KB media. After 7 days, media was removed, tubes were washed three times with 1 mL of deionised H_2_O. H_2_O was removed and 1 mL of 0.05% crystal violet was added to each tube, incubated for 2 min at room temperature and then washed three times with 1 mL of deionised H_2_O. The stain was eluted with 95% ethanol for 30 min, after which tubes were vortexed, and 100 μL was used to measure OD_570_ in a plate reader. Increased cellular adherence corresponded to larger OD values. Each clone was measured in triplicate.

### 2.6 DNA Sequencing

DNA was extracted from clones obtained from both ancestral *E. coli* and *P. fluorescens* types, as well as from evolved populations under different experimental conditions. For monoculture‐evolved *E. coli* , one LCV clone and one SCV clone were selected from each of the five replicate populations (E1–E5). For co‐culture‐evolved *E. coli* , one clone was selected from each of the five replicate populations (C1–C5). For monoculture‐evolved *P. fluorescens* , one WS clone and one SM clone were selected from each of the five replicate populations (P1–P5). Similarly, for co‐culture‐evolved *P. fluorescens* , one WS clone and one SM clone were selected from each of the five replicate populations (C1–C5). DNA was extracted using a GenElute Bacterial Genomic DNA kit (Sigma‐Aldrich). Whole genome sequencing was conducted using Azenta's next‐generation sequencing service, which obtained paired‐end 150 bp reads using an Illumina Novaseq 6000. The minimum average read coverage per population for *E. coli* was ~200‐fold, and for *P. fluorescens* ~150‐fold. All reads with a quality score less than Q30 were removed from our analysis. Sequence data were assembled to reference genomes using Breseq (Deatherage and Barrick 2014). By sequencing the ancestral clones, we were able to identify mutations present in the ancestor relative to the reference assembly and as such could identify de novo mutations in evolved clones—the *P. fluorescens* *SBW25* reference genome GenBank accession number is AM181176. The *E. coli MG1655* GenBank accession number is NC_000913. Coverage plots were made by mapping all reads onto the recipient genome using the BWA‐MEM package (Li and Durbin 2009); the resulting SAM file converted to a BAM file, and then SAMtools depth was used to calculate the depth at each base pair in this bam file (Li et al. 2009). The depth at each nucleotide was normalised by the average depth across the whole genome, and plots were made with 500 bp bins using the R package ggplot2. Structural breakpoints (duplication in co‐culture LCV *E. coli* for example) were confirmed using GRIDSS software (Cameron et al. 2017).

### 2.7 Hypergeometric Test for Multi‐Hit Genes

We determined whether mutations in a given gene were more likely than chance to be associated with one of the colony types or experimental treatments. First, we created a list of multi‐hit genes, defined as genes containing de novo mutations in at least three replicates; these six genes are displayed in Figure 3B. Statistical significance of the enrichment of a mutation within the subset of clones was assessed using the hypergeometric distribution. For this test, the response variable was the observed number of mutations in a specific clonal group, and the predictor variable was the total number of clones in the relevant experimental treatment or colony type. The test assesses whether the observed number of mutations is significantly different from what would be expected based on the overall proportion in the entire subset of clones. *p* values were adjusted for multiple comparisons using the Bonferroni correction (accounting for 1 test for *E. coli* and 2 tests for *P. fluorescens* ). For *P. fluorescens* , we evaluated whether mutations in PFLU_3409 were associated with the SM variant, whether mutations in PFLU_3409 occurred more frequently in monoculture than in co‐culture, and whether mutations in the PFLU_1661 gene occurred more frequently in co‐culture than in monoculture.

### 2.8 Amplicon Sequencing Fitness Assay

To investigate the interaction between *E. coli* and *P. fluorescens* , we conducted a competitive fitness assay and quantified differences in population proportions over time using long‐read sequencing of 16S amplicons. Two types of microbial mixtures (master mixes) were prepared, in which the focal *E. coli* was present at either high or low proportions in a *P. fluorescens* background. Each condition was replicated six times, with the master mixes inoculated into 3 mL of KB media following the experimental evolution setup. Genomic DNA (gDNA) was extracted from the master mixes on day 0 and from the microcosms after 7 days of incubation using the GenElute Bacterial Genomic DNA Kit (Sigma‐Aldrich).

To differentiate between *E. coli* and *P. fluorescens* , the 16S ribosomal DNA (rDNA) region was amplified using universal bacterial primers (Forward: GATCMTGGCTCAGRWTGAACS; Reverse: TATTCCCYACTGCTGCCTC). PCR products were purified with the Monarch Spin PCR & DNA Cleanup Kit and sequenced on the Nanopore platform. Sequencing libraries were prepared using the Rapid Barcoding Kit (Oxford Nanopore Technologies) following the manufacturer's instructions. Base calling was performed on POD5 files using Dorado version 0.8.0 (https://github.com/nanoporetech/dorado) with the latest SUP model (dna_r10.4.1_e8.2_400bps_sup@v5.0.0). Demultiplexing was conducted using Dorado Demux, and barcode and adapter sequences were trimmed using Porechop (https://github.com/rrwick/Porechop).

We analysed the amplicon sequencing data using a custom Python pipeline. Reads were aligned to reference sequences with Minimap2, and only alignments with a score ≥ 50 were retained. Reads with ambiguous or tied alignments were excluded from the final analysis. Species proportions were calculated by determining the number of reads uniquely aligned to each reference sequence and expressing these counts as a proportion of the total aligned reads. The amplicon sequencing analysis pipeline is available on GitHub (https://github.com/AyshaSezmis/amplicon‐analysis‐pipeline). PFLU_1661 Gene Mutant Assay: The same pipeline was used to assess the performance of two *P. fluorescens* mutants containing mutations in the PFLU_1661 gene. These mutants, which were selected as SM clones from co‐culture populations 1 and 2, were chosen because they lacked WS mutations that are known to be adaptive in structured, mixed‐species environments (Workentine et al. 2013). Mutants were mixed at low proportions with ancestral *P. fluorescens* and *E. coli* in two separate experimental setups, where *E. coli* was present at either high or low proportions. Each condition was replicated six times.

To distinguish between ancestral strains and mutants, we performed two separate PCR amplifications: one using the 16S primers (Forward: GATCMTGGCTCAGRWTGAACS; Reverse: TATTCCCYACTGCTGCCTC) to differentiate *E. coli* from *P. fluorescens* and the other using primers specific to the *PFLU_1661* gene region (Forward: TTAATGTCTCTGGCGCAACC; Reverse: AGCTGTAGATCCAGCTGAG). These primers specifically amplify *P. fluorescens* DNA and do not target *E. coli* .

Sequencing of PCR products was performed on the Nanopore platform, and subsequent analysis was carried out as described for the 16S amplicon sequencing. Reads were aligned, filtered and analysed using the same custom Python pipeline to quantify the relative proportions of ancestral strains and *PFLU_1661* mutants in the final populations.

Fitness differences between the focal strain and the reference strain were calculated by measuring the rate of change in the natural log (LN) ratio of the focal to reference strain over time (Waser 2000). For *P. fluorescens* , the focal strain consisted of PFLU_1661 mutants, and the reference strain was the *P. fluorescens* ancestor (as shown in Figure 4A,B). For *E. coli* , the focal strain was the *E. coli* population, and the reference strain was the *P. fluorescens* population (as shown in Figure 4C). This approach allowed for the quantification of relative fitness differences or capacity for the focal strain to increase in frequency in the conditions of the co‐culture assays.

### 2.9 Statistical Methods

Ancestral versus week 8 population compositions were assessed with two proportion *z*‐tests and chi‐square tests. *P. fluorescens* frequency dependence was assessed with unpaired *t*‐tests. Motility and biofilm assays were assessed with one‐way ANOVA, followed by post hoc Tukey's multiple comparisons. Statistical tests were conducted in *GraphPad Prism*; coverage plots were generated in *R*.

## 3 Results

### 3.1 *E. coli* Evolves a Second Type in Monoculture, But Not in Co‐Culture

**FIGURE 1:**
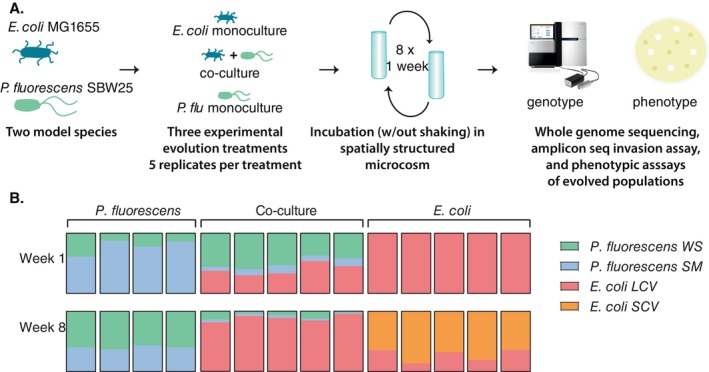
(A) Overview of evolution experiment and analysis. (B) Relative abundance of different *E. coli* and *P. fluorescens* types in each experimental microcosm at the beginning and end of an 8‐week evolution experiment. Community composition changed in all treatment groups after 8 weeks of evolution. WS and SM evolved in every replicate *P. fluorescens* monoculture and co‐culture population. WS frequency increased in the *P. fluorescens* group. A small colony variant (SCV) evolved in each of the *E. coli* monoculture replicate populations. Co‐culture communities were dominated by *E. coli*.

To study the impact of co‐culture on the outcomes of *P. fluorescens* WS evolution, we propagated replicate populations of *E. coli* MG1655 and *P. fluorescens* SBW25 in either monoculture or co‐culture (Figure 1A).

In the *P. fluorescens* monoculture group, we confirmed that *P. fluorescens* diverged into the WS and SM types in all replicate populations after 1 week of evolution (Figure 1B). After 8 weeks of evolution, WS and SM types, as well as the WS mat, were still present. The proportion of WS increased during the course of the experiment, with WS making up an average of 22.375% ± 11.96% of total colonies in each population after 1 week, but 60.2% ± 2.69% after 8 weeks. The overall change across populations was significant by a two‐proportion *z*‐test (*Z* = 5.006, *p* < 0.0001, *n* = 4) (Figure 1B, Supporting Information).

Similar to *Pseudomonas* species, *E. coli* is capable of evolving biofilm‐forming colony types, such as the red, dry, rough phenotype (Hufnagel et al. 2015; Uhlich et al. 2006). In the *E. coli* monoculture treatment, we hypothesised that *E. coli* would adapt to long‐term growth in the static microcosm and diverge into multiple co‐existing types. We observed that a new *E. coli* colony type evolved that appeared smaller, raised, and brighter than the ancestral colonies. This colony type evolved in all five replicate microcosms and, on average, in each *E. coli* monoculture microcosm comprised an average of 27.2% ± 10.16% (Figure 1B). However, these types did not form mats at the air–liquid interface. We confirmed that this SCV phenotype was heritable by re‐streaking a number of SCV colonies and finding that these colonies always produced more small colonies. Other evolution experiments involving *E. coli* have also reported the evolution of small colony variants (Saxer et al. 2010); these colonies often constitute slow‐growing sub‐populations, with glossy convex surfaces (Paratore et al. 2018). Henceforth, we refer to the two colony types observed in *E. coli* monoculture populations as the SCV and the LCV, which appeared similar to the *E. coli* ancestor.

Next, we looked at co‐culture microcosms and found that WS dominated after 1 week, with the average composition of each culture: WS 49.66% ± 9.67%, SM 10.44% ± 2.72%, *E. coli* 39.96% ± 9.43%. After 8 weeks of growth, in all five replicate microcosms, *E. coli* were the most numerous type: WS 7.95% ± 5.05%, SM 3.58% ± 1.15%, *E. coli* 87.17% ± 5.5%; the changes in composition from week 1 to week 8 were significant by *Χ* ^*2*^ test (*Χ* ^*2*^_2,5_ = 57.37, *p* < 0.0001). In co‐culture, surface mats were observed after 8 weeks of growth, suggesting that the air–liquid interface was occupied in these cultures. We did not observe *E. coli* SCV in co‐culture microcosms, and on an agar plate, co‐culture *E. coli* appeared similar to the ancestor and the LCV in monoculture. The repeated evolution of the *E. coli* SCV in each of five replicate monoculture populations, but not in co‐culture, suggests that similar selection pressures act on *E. coli* in each replicate population, but the outcomes of selection are different in the presence of *P. fluorescens* .

### 3.2 Phenotypes of Evolved Clones

**FIGURE 2:**
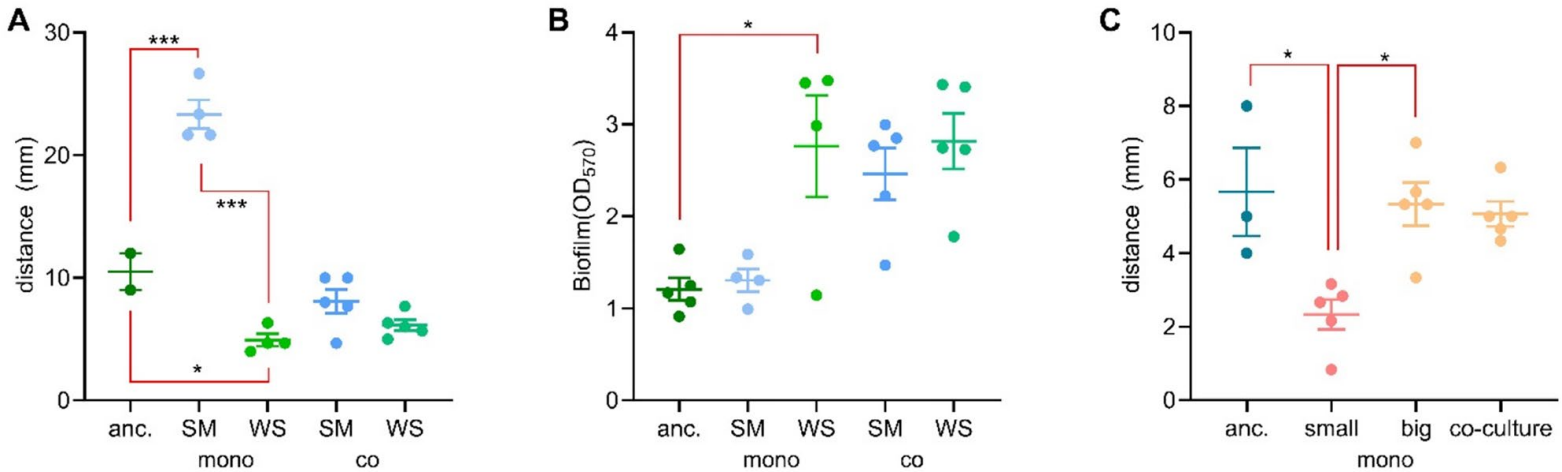
Motility (A) of ancestral and evolved *P. fluorescens* ; motility measurements show the mean of three technical replicates of clones of each type from the replicate evolved microcosms. Biofilm forming capacity (B) of ancestral and evolved *P. fluorescens* ; each point is a clone taken from a replicate microcosm. The horizontal lines show the mean across evolution experiment replicates. Motility of ancestral and evolved *E. coli* (C) (Methods) *, **, and *** indicate *p* values < 0.05, < 0.005 and < 0.0001, respectively. Error bars are the standard error of the mean. Significance was calculated using one‐way ANOVA followed by post hoc Tukey's with multiple comparisons.

We examined the niche‐specific phenotypes of *P. fluorescens* WS and SM to determine whether *E. coli* types showed similar phenotypic differentiation and how co‐culture treatment affected these phenotypes. In *P. fluorescens* monoculture, we found similar trade‐offs between biofilm formation and motility observed in other *Pseudomonas* species (Ueda et al. 2020; Caiazza Nicky et al. 2007). *P. fluorescens* SM clones that evolved in monoculture conditions were significantly more motile than both the ancestor and WS (one‐way ANOVA with post hoc Tukey's multiple comparison *f* = 71.93, *p* < 0.0001) (Figure 2A, Supporting Information). Similarly, we confirmed previous findings that *P. fluorescens* WS have reduced motility compared to ancestral *P. fluorescens* (one‐way ANOVA with post hoc Tukey's multiple comparison *f* = 71.96, mean difference = 5.588, SE = 1.538, *p* = 0.0177) (Pentz and Lind 2021) (Figure 2A), and increased biofilm‐forming capacity (Guttenplan and Kearns 2013) (Figure 2B). While *E. coli* SCV had reduced motility compared to the LCV *E. coli* cells (one‐way ANOVA with post hoc Tukey's multiple comparison *f* = 7.196, mean difference = 3.002, SE = 0.7784, *p* = 0.0084) (Figure 2C), they did not form mats.

In co‐culture, the dominant *E. coli* type did not drive out *P. fluorescens*, although the population size of *P. fluorescens* was lower in co‐culture than in monoculture ( *P. fluorescens* monoculture 1.05 ± 0.164 × 10^9^, coculture; 1.56 ± 0.635 × 10^8^ CFU/mL). When we compared the motility of the SM from monoculture and co‐cultures, we noted that co‐culture SM types were not motile (one‐way ANOVA with post hoc Tukey's multiple comparison *f* = 71.93, mean difference = 15.26, SE = 1.191, *p* < 0.0001), suggesting that they share phenotypic features with WS and that co‐culture with *E. coli* may have altered the evolutionary trajectory of the WS and SM types.

### 3.3 Genetic Causes of the Big and Small Colony Variants of *E. coli*

We carried out whole genome sequencing to determine the genetic causes of adaptation in the monoculture and co‐culture treatments. We obtained genome sequences for *E. coli* and *P. fluorescens* clones isolated from each replicate microcosm after 8 weeks of evolution. In the *E. coli* populations, relatively few de novo SNPs were observed, with approximately two mutations detected per clone, although many clones had large duplications encompassing many genes. In contrast, more mutations were found in *P. fluorescens* , with approximately 15 mutations per clone. In addition, clones from two of the monoculture and two co‐culture evolved *P. fluorescens* populations had a mutation in *mutL*, a known mutator gene (Pal et al. 2007), and were found to have more new mutations than non‐*mutL* mutants (Supporting Information).

**FIGURE 3:**
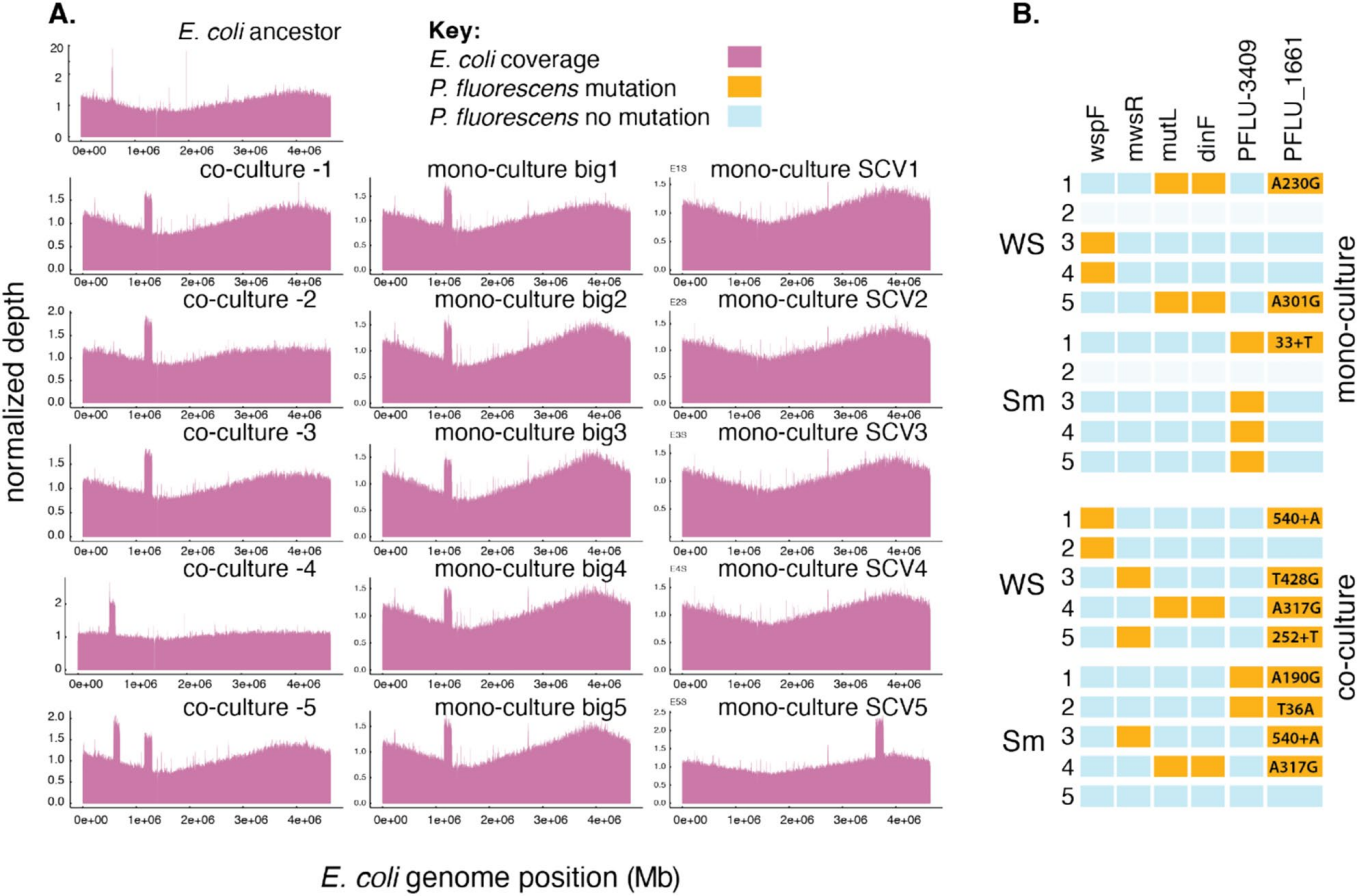
The genetic causes of *E. coli* and *P. fluorescens* adaptation in static microcosms. (A) Coverage plots for the *E. coli* ancestor and *E. coli* co‐culture and monoculture evolved populations. SCV refers to the small colony variants identified in all monoculture‐evolved *E. coli* populations. (B) Multi‐hit genes in *P. fluorescens* populations. The *mwsR* and *wspF* genes are known targets for WS mutations. The *mutL* mutations were not associated with any treatment. Only genes that evolved mutations in more than three replicate populations (multi‐hit genes) are shown. The nucleotide changes for PFLU_1661 are shown to highlight the independent evolution of PFLU_1661 mutations in SM and WS isolated from the same microcosm. Note that replicate 2 of the *P. fluorescens* monoculture treatment was excluded due to contamination.

We then looked for genetic parallelism, the evolution of mutations in the same gene across independently evolved populations, to provide evidence for natural selection. We started by looking at the *E. coli* LCV and SCVs. No SNPs or small indels were found to be in more than two replicate populations (Supporting Information). However, we found that the five LCV *E. coli* clones isolated from each of the replicate monoculture *E. coli* populations, and four out of five *E. coli* clones isolated from the co‐culture populations had a large duplication within a region of the *E. coli* MG1655 genome, spanning between nucleotide positions 1,156,000—1,293,000 (Figure 3A). The four co‐culture *E. coli* clones had a duplication of 147–150 genes starting at *holB* and including all genes up to either *rssA*, *rssB* or *hns*. Two of the monoculture *E. coli* LCV clones (E1 and E5) had a similar duplication, while the other three monoculture *E. coli* clones had multiple smaller duplications that fell within the range of the larger 150 gene duplication; for example, the LCV from populations E3 contained a 50,050 bp duplicated region from position 1,159,561, a 19,280 bp duplication from position 1,221,431 and a 12,430 bp duplication from position 1,253,531. The genes found within this duplicated region are available in the Supporting Information. The parallelism and strong association of this duplication with one particular clone phenotype (LCV, hypergeometric distribution, *p* = 0.002) support that the evolutionary spread of the duplication was driven by natural selection. Duplications of this region were absent from SCVs. We found no evidence for an evolved genetic change that was specific to the *E. coli* SCV (Figure 3A).

### 3.4 Parallel Evolution Suggests Co‐Culture‐Specific Adaptations in *P. fluorescens*

In *P. fluorescens* monoculture and co‐cultures, sequencing confirmed the presence of mutations known to cause the WS phenotype (Spiers et al. 2003; McDonald et al. 2011; Ardré et al. 2019), for two clones selected, P1W and P5W, no known WS mutations were identified despite a clear wrinkly phenotype on solid and liquid media (Figure 3B). This absence was confirmed by manual examination of the alignment of sequence reads to known WS genes (*mwsR* and the *aws* and *wsp* operon) in the Integrative Genomics Viewer (Robinson et al. 2011). In addition to the WS‐specific mutations, we identified a significant association between mutations in PFLU 3409 (a methyl accepting chemotaxis protein) and the monoculture ancestral *P. fluorescens* colony phenotype (hypergeometric distribution, *p* = 0.005) (Figure 3B).

We also identified parallel evolution of the gene PFLU_1661 in *P. fluorescens* isolated from co‐cultures more often than in *P. fluorescens* isolated from monocultures (hypergeometric test, *p* = 0.032), labelled as RS_0816 in NCBI reference sequence NC_012660. This gene has appeared in a previous transposon mutagenesis study designed to identify genes essential for the WS phenotype; in other annotations in that study, mutants were found to form mats at the surface of the microcosm (McDonald et al. 2009). In this study, mutations in the PFLU_1661 gene were found in co‐culture evolved WS and smooth populations, but not in monoculture evolved populations, suggesting that mutations in this gene confer a benefit only in co‐culture with *E. coli* . Mutations in the PFLU_1661 gene evolved in both the WS and SM phenotypes—11 out of the 18 sequenced *P. fluorescens* clones, and 8/10 of the clones isolated from co‐cultures (both WS and SM), evolved mutations in this gene. This result suggests that this adaptation was not specific to the WS or SM phenotypes (hypergeometric test, *p* = 0.31).

**FIGURE 4:**
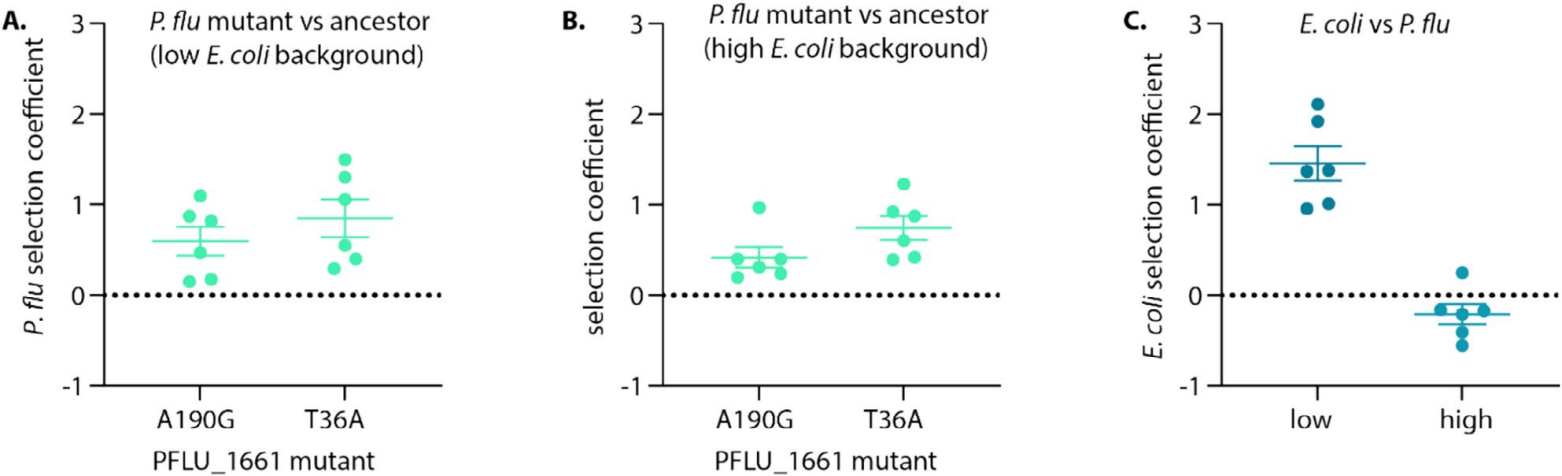
Invasion dynamics of *P. fluorescens* PFLU_1661 mutants and *E. coli* ancestor in co‐culture. (A, B) Selection coefficients of two *P. fluorescens* mutants, each carrying mutations in the *PFLU_1661* gene (A190G and T36A). Both mutants show positive selection coefficients, indicating a capacity to invade the ancestral *P. fluorescens* genotype. Panel (A) represents co‐cultures where *E. coli* was initially at a low frequency (~40%), while panel (B) shows co‐cultures with *E. coli* at a high frequency (~80%). The results demonstrate that both *PFLU_1661* mutants can invade the ancestral *P. fluorescens* population regardless of the initial *E. coli* ancestor frequency. (C) Selection coefficients of *E. coli* in co‐culture with *P. fluorescens* . *E. coli* displays a positive selection coefficient when starting at a low frequency (~40%), indicating the capacity to invade from a low relative frequency. In contrast, when *E. coli* starts at a high frequency (~80%), its selection coefficient is slightly negative or indistinguishable from zero, indicating no significant invasion advantage. Error bars represent standard deviation of the mean.

To further test the fitness consequences of PFLU_1661 mutations, we conducted competitive fitness assays using amplicon sequencing (Methods). In co‐culture experiments with *E. coli* that included the *P. fluorescens* wildtype, both PFLU_1661 mutants (A190G and T36A) exhibited positive selection coefficients relative to the ancestral *P. fluorescens* (Figure 4A). Importantly, the fitness advantage of these mutants was similar regardless of the initial *E. coli* frequency (Figure 4B). In contrast, *E. coli* fitness was dependent on its starting frequency (Figure 4C). *E. coli* exhibited a strong positive selection coefficient when starting at a low frequency (~40%) but showed no significant fitness advantage, and even a slight negative trend, when starting at a high frequency (~80%). These results confirm that mutations in PFLU_1661 confer a selective advantage to *P. fluorescens* in co‐culture with *E. coli* .

## 4 Discussion

The genetic changes and evolutionary forces that drive the WS adaptive radiation are one of the best‐studied examples of microbial laboratory adaptation (Rainey and Travisano 1998; Bantinaki et al. 2007; McDonald et al. 2009; Lind et al. 2015, 2017; Spiers et al. 2003; Moshynets et al. 2022; Rainey and Rainey 2003; Koza et al. 2017). The WS radiation also provides insights into microbial ecology, showing how a structured, multi‐niche environment can lead to the stable maintenance of multiple co‐existing types (Fukami et al. 2007). However, the colonisation of newly available niches in natural environments is quite likely to involve more than a single species (McDonald 2019). Here, we investigated the adaptive radiation of *P. fluorescens* together with the well‐studied bacterial species, *E. coli* MG1655, which is also known to evolve multiple co‐existing types in a range of experimental evolution conditions (Good et al. 2017; Cooper and Lenski 2010; Rosenzweig et al. 1994). Previous studies have explored the adaptive radiation of *Pseudomonas fluorescens* in the context of interspecific competition. For example, Gómez and Buckling (2013) found that natural microbial communities constrained *P. fluorescens* diversification by reducing available niches, while Jousset et al. (2016) demonstrated that competition between closely related *P. fluorescens* strains promoted diversification (Jousset et al. 2016; Gómez and Buckling 2013). More recently, Chu et al. (2021) showed that increasing the diversity of interspecific competitors enhanced morphological diversity in *P. fluorescens* , driven by niche‐specific competition (Chu et al. 2021). Here, we focus on a two‐species experiment with *Escherichia coli* , building on previous results by focusing on both species in a two‐species experiment and identifying some of the genetic causes of adaptation.

### 4.1 The *E. coli* Small Colony Variant Evolved and Persisted in Monoculture Evolved Populations But Not in Co‐Culture

First, our results show that *E. coli* diversifies into two coexisting types that could be distinguished by colony size and that persisted for eight transfers. While we found that the SCV was genetically distinct from the LCV, we did not discover the genetic cause of the SCV. A previous evolution experiment by Saxer, Doebeli and Travisano found that small colony variants are novel resource specialists, filling a niche in an environment depleted of glucose (Saxer et al. 2010). Similarly, Rozen and Lenski propose that observed small colony variants may be able to use metabolites excreted during glucose metabolism by the big colony type (Rozen and Lenski 2000). It is possible that in depleted media—as would occur after 7 days of growth—there is a niche for a slow‐growing *E. coli* that utilises alternative carbon sources. While *P. fluorescens* quickly evolved subpopulations of SM and WS types, *E. coli* was slower to diversify—the two types were not evident after 1 week of evolution. Finally, despite the use of *P. fluorescens* growth conditions (KB and 28°C), *E. coli* evolved to be the dominant type in co‐cultures. We hypothesised that this may be because *E. coli* is a facultative anaerobe and can outperform *P. fluorescens* , which has greater oxygen requirements (Koza et al. 2011).

### 4.2 Monoculture and Co‐Culture Specific Adaptations in *P. fluorescens*

Previous studies have identified genetic variants associated with WS adaptation (McDonald et al. 2009; Lind et al. 2015) and microcosms that fluctuate between shaken and static conditions during experimental evolution (Beaumont et al. 2009; Gallie et al. 2019). However, adaptation to the liquid column—the niche of the *P. fluorescens* SM type—has been less well studied. One of the well‐established mechanisms of adaptation to the air–liquid interface, or mat formation, is the evolution of a permanent biofilm state, by the increased production of the secondary signalling molecule cyclic di‐GMP (Goymer et al. 2006). The phenotype is achieved by mutations that result in the activation of GGDEF domain proteins, and most (but not all) WS evolve by mutations that disrupt the regulation of AwsR (negatively regulated by AwsX), WspR (negatively regulated by WspF) and MwsR (McDonald et al. 2009). We found that most of our WS were caused by mutations in the *wspF* and *mwsR* genes, although we were unable to discover the causal mutation for three WS. In addition to these loci known to be associated with adaptation to static microcosms, we identified two new loci. First, we found that many SM types had mutations in PFLU_3409, a methyl sensing chemosensory protein (Salah Ud‐Din and Roujeinikova 2017; Galperin 2009). The repeated selection of mutations in this gene supports that the *P. fluorescens* population that resides in the liquid column is adapting in response to different selection pressures than the WS.

Second, we found evidence for a beneficial mutation that evolved more often in *P. fluorescens* populations growing in co‐culture with *E. coli* than *P. fluorescens* populations in monoculture. Mutations that inactivated PFLU_1661 were recovered from two WS and one SM that evolved in monoculture conditions. In contrast, *P. fluorescens* in all co‐cultures evolved mutations in this gene, and three co‐cultures evolved mutations in both WS and SM. PFLU_1661/RS_0816 is predicted to encode a 214 amino acid sugar transferase (Pal et al. 2019). It is noteworthy that in two of the co‐culture *Pseudomonas* populations, the WS and SM types from the same microcosm independently evolved different genetic variations in the PFLU_1661 gene (Figure 3B). This suggests that niche‐specific selection was strong enough to prevent the first PFLU_1661 mutation that evolved (in either a WS or SM type) from fixing within the *P. fluorescens* population. Indeed, in our invasion experiments that were inoculated with ancestral *E. coli* , ancestor *P. fluorescens* and the PFLU_1661 mutants, WS types still readily evolved. However, other evolutionary dynamics could have been behind the more rapid evolution of PFLU_1661 mutants in co‐cultures. For example, if the *E. coli* sped up the rate of the WS adaptive radiation, this could partially explain the delayed evolution of the PFLU_1611 mutations in the monocultures.

Altogether, these results provide phenotypic and genetic evidence that supports the presence of another species altering the outcomes of the *E. coli* and *P. fluorescens* adaptive diversification. An interesting, repeatable result of microbial experimental evolution studies with a single focal species has been the evolution of diverse, coexisting types, even in simple laboratory conditions (Rainey and Travisano 1998; Frenkel et al. 2015; Good et al. 2017; Treves et al. 1998). This work suggests that this finding may not generalise to natural systems with multiple species.

## Author Contributions

**Gareth Howells:** investigation, writing – original draft, methodology, writing – review and editing, formal analysis, data curation, visualization, validation. **Aysha L. Sezmis:** methodology, validation, software, formal analysis, investigation. **Christopher Blake:** methodology, formal analysis. **Michael J. McDonald:** conceptualization, funding acquisition, writing – review and editing, visualization, project administration, supervision, resources, investigation.

## Conflicts of Interest

The authors declare no conflicts of interest.

## Supporting information


Data S1.

## Data Availability

Assembled genomes and raw DNA sequence files have been deposited at NCBI Bioproject PRJNA988163. Code and instructions for the amplicon sequencing pipeline are available at github (https://github.com/AyshaSezmis/amplicon‐analysis‐pipeline). Supporting Information link: DOI: 10.6084/m9.figshare.28340420.
